# Optimization of High Temperature and Pressurized Steam Modified Wood Fibers for High-Density Polyethylene Matrix Composites Using the Orthogonal Design Method

**DOI:** 10.3390/ma9100847

**Published:** 2016-10-18

**Authors:** Xun Gao, Qingde Li, Wanli Cheng, Guangping Han, Lihui Xuan

**Affiliations:** College of Material Science and Engineering, Northeast Forestry University, Harbin 150040, China; dongbeilinyedaxue211@hotmail.com (X.G.); liqingde@gmail.com (Q.L.); guangping.han@nefu.edu.cn (G.H.); leeh91@hotmail.com (L.X.)

**Keywords:** high temperature-pressurized steam, orthogonal design method, interfacial compatibility, surface treatment, composite material

## Abstract

The orthogonal design method was used to determine the optimum conditions for modifying poplar fibers through a high temperature and pressurized steam treatment for the subsequent preparation of wood fiber/high-density polyethylene (HDPE) composites. The extreme difference, variance, and significance analyses were performed to reveal the effect of the modification parameters on the mechanical properties of the prepared composites, and they yielded consistent results. The main findings indicated that the modification temperature most strongly affected the mechanical properties of the prepared composites, followed by the steam pressure. A temperature of 170 °C, a steam pressure of 0.8 MPa, and a processing time of 20 min were determined as the optimum parameters for fiber modification. Compared to the composites prepared from untreated fibers, the tensile, flexural, and impact strength of the composites prepared from modified fibers increased by 20.17%, 18.5%, and 19.3%, respectively. The effect on the properties of the composites was also investigated by scanning electron microscopy and dynamic mechanical analysis. When the temperature, steam pressure, and processing time reached the highest values, the composites exhibited the best mechanical properties, which were also well in agreement with the results of the extreme difference, variance, and significance analyses. Moreover, the crystallinity and thermal stability of the fibers and the storage modulus of the prepared composites improved; however, the hollocellulose content and the pH of the wood fibers decreased.

## 1. Introduction

Wood plastic composites (WPCs), a new type of environmentally-friendly material involving the use of cellulose fibers as reinforcing agents in composite materials based on polymeric matrices, have already been widely utilized in various fields, such as architecture and parquet. They have recently attracted a growing interest, as shown by the large number of articles and reviews published during the past decade [1,2]. Compared to glass, cellulose offers a number of advantages, such as low density, which leads to lighter material; renewable and ubiquitous character; nonabrasive nature, causing less wear to the mixing and molding equipment; higher flexibility, which reduces the risk of fragmentation during processing; and, most importantly, its low cost [3]. Nonetheless, many technical problems associated with WPCs still exist. For instance, inferior interfacial compatibility between the wood fibers and the plastic, as well as poor dispersion of the wood fibers [4] and the formation of bottlenecks, restrict further enhancement of the physical and chemical properties of WPCs. In order to improve the interfacial compatibility, a method for the chemical modification of the wood fibers, for e.g., alkaline treatment [5], acylation [6,7], etherification [8], or graft copolymerization [9], is generally employed, and the mechanical properties of the composites modified by all of these conventional treatments increase by 10%–35%, compared to the untreated composites. This improvement is attributed to the elimination of impurities due to treatment and introduction of compatible molecular structure onto the wood fiber surfaces. However, the chemical modification of the wood fibers usually exhibits several disadvantages, such as high cost or complexity of the modification process, and environmental pollution caused by the processing methods.

Steam treatment is a common and environmentally-friendly method. It has been widely used to modify solid wood [10,11], particleboard [12], fiberboard [13], and wheat straws [14] to improve dimensional stability, decrease hygroscopicity, and decrease the ash content of wheat straws, which further enhances their physical and mechanical properties. However, steam modification to improve the surface bondability between wood fibers and matrix resin was rarely studied; especially, the research of high temperature and pressurized steam treatment is much less. In this study, exposing the wood fibers to a high temperature and pressurized steam treatment can induce degradation of the semi-cellulose with a poorer thermal stability [15,16], leading to a significantly lower number of free hydroxyl groups on the fiber surface, decreased surface polarity, increased surface roughness, and higher crystallinity of the cellulose [17], which further improves the interfacial bondability between the fibers and the plastic matrix and, therefore, enhances the mechanical properties of WPCs [18,19]. The orthogonal design method efficiently deals with multifactor design and screening optimum levels by using the orthogonal design table [20,21]. The main objective of this study was to modify the wood fibers via different temperature and pressurized steam treatments using the orthogonal design method [22] to determine the optimum parameters for the steam modification process. This study, in particular, aimed at improving the dispersion of the poplar fibers in a high-density polyethylene (HDPE) melt to enhance the interfacial bondability between the two components. Furthermore, the modification mechanism was described, and its effect on the mechanical properties of the fabricated WPCs was comprehensively investigated.

## 2. Material and Methods

### 2.1. Raw Materials

Commercially available poplar veneers were purchased from Yong Xv Industrial Wood Composites Factory (Harbin, China). High-density polyethylene (HDPE) (density 950 kg·m^−3^ and melt flow index 1.95 g/10 min) was provided by Daqing Petrochemical Co. (Daqing, China). Industrial-grade paraffin, acetone, sodium chlorite, ethanol, and glacial acetic acid (Quanxi Chemical Co., Ltd., Nanjing, China) were used as received without further purification. 

### 2.2. Modification of Wood Fibers

The poplar fibers were subjected to high temperature and pressurized steam generated by a steam generator at different pressures and steam temperatures. After exposure to the steam for 5–20 min, the samples were placed in a drying oven at 105 °C to decrease the moisture content to about 3%–5%.

The orthogonal experimental design is a popular method to deal with the experiments, including multiple factors and levels [23]. It has been successfully applied to several fields, and it saves a large amount of time for acquiring the optimum level group [24,25]. For the high temperature and pressurized steam modification of wood fibers, three parameters were mainly considered, i.e., the steam temperature (120, 140, 160, and 170 °C), the steam pressure (0.2, 0.4, 0.6, and 0.8 MPa), and the processing time (5, 10, 15, and 20 min). An orthogonal array of three factors and four levels, as listed in Table 1, was determined to investigate the effects of modification on the tensile, flexural, and impact strengths of the HDPE composites reinforced with modified poplar fibers. According to the factors and levels listed in Table 1, an L16 (45) orthogonal experimental design listed in Table 2 was obtained, where sixteen experimental conditions were conducted [26]. 

### 2.3. Characterization of Wood Fibers 

#### 2.3.1. Hollocellulose Extraction

The sodium chlorite method was used to measure the hollocellulose content according to the GB/T 2677-1995 standard [27]. A mixture of toluene and ethanol was used to extract the substance from the wood fibers, and then sodium chlorite and glacial acetic acid were added to the extracted substance for the delignification process. After washing, the extracted wood fibers were dried at 105 °C in a drying oven until the weight remained constant. The hollocellulose content was calculated based on the oven-dried weight.

#### 2.3.2. The pH Value

Three grams of wood fibers were weighed using an analytical balance and placed in a beaker. Then, 30 mL of distilled water, freshly boiled and cooled to room temperature, was added. The mixture was further thoroughly stirred. The pH of the wood fibers was measured according to the GB/T 6043-1999 standard [28]. 

#### 2.3.3. Fourier Transform Infrared (FTIR) Spectroscopy

FTIR spectroscopic measurements were performed using a Nicolet 6700 FTIR spectrometer (Thermo Fisher Scientific Co., Ltd., Waltham, MA, USA) in the range 3500–400 cm^−1^ with a scanning rate of 32 scans per minute. The resolution for the spectra was 4 cm^−1^. All of the steam-modified fiber samples were dried in an oven at 40 °C for 48 h and cooled to 20 °C before placing them in the sample chamber. Three replicated measurements were recorded for each condition.

#### 2.3.4. Wide-Angle X-ray Diffraction (WXRD)

Wide-angle X-ray diffraction (WXRD) patterns of the fiber samples before and after modification were obtained using a D/MAX 2200 X-ray diffractometer (Rigaku Corporation, Sendagaya, Japan). Prior to the measurement, the sample was placed onto the supporter and compactly pressed. The WXRD data were generated by a diffractometer with CuKα radiation (λ = 1.542 Å) at 40 kV and 30 mA over the angular range 2θ = 5°–40°, and a step size of 5°·min^−1^. The degree of crystallinity or crystallinity index (*CI*, %) for each sample was evaluated by using Equation (1):

(1) $$CI=\frac{I_{002}-I_{\mathrm{am}}}{I_{\mathrm{am}}}$$

where *I*_002_ is the maximum diffractive strength of the 002 crystalline plane and *I*_am_ is the diffractive strength of the non-crystalline plane.

#### 2.3.5. Thermogravimetric Analysis (TGA)

The weight loss during the pyrolysis of the poplar wood fibers was assessed by using a TA 309F3 thermal gravimetric analyzer (TA Instruments, New Castle, DE, USA). Samples around 5 mg were heated from 20 to 600 °C at a heating rate of 10 °C·min^−1^ under a constant nitrogen flow of 30 mL·min^−1^.

### 2.4. Preparation of Wood Fiber/HDPE Composites

To prepare the wood fiber/HDPE composites, the wood fiber samples were first placed in an oven and dried at 105 °C to decrease the moisture content to 3%–5%. The wood fibers, HDPE, and paraffin were then mixed in a 50:48:2 ratio and then stirred in a high-speed mixer. The uniform mixture was subsequently placed in a two-stage plastic extruder (SJSH30/SJ45, Nanjing Rubber and Plastic Mechanic Co., Ltd., Nanjing, China) with a rotation speed of 60 rpm. The temperatures of the twin-screw extruder were controlled at 160, 165, 170, 170, 185, 170, and 165 °C from the feeding zone to the die, respectively; however, those of the single-screw extruder were 150 (feeder), 155, 165, 165, and 155 °C (die), respectively.

### 2.5. Characterization of the Prepared Wood Fiber/HDPE Composites

#### 2.5.1. Mechanical Properties

Five specimens were randomly selected for each mechanical test. Samples for flexural, tensile, and impact tests were prepared with sizes of 80 mm × 13 mm × 4 mm, 165 mm × 20 mm × 4 mm, and 80 mm × 10 mm × 4 mm, respectively. Mechanical properties of the samples were tested using an RGT-20A electronic mechanics testing machine (Shenzhen REGER Instruments Co., Ltd., Shenzhen, China). The flexural, tensile, and impact tests were conducted according to the GB/T 9341-2000 test, the GB/T 1040-1992 test, and the GB/T 1043-1993 test [29], respectively. The final measurements were obtained by taking the arithmetic mean of the five specimens. 

Dynamic mechanical analysis (DMA) of the composites was performed using a TA Q800 analyzer (TA Instruments Inc., New Castle, DE, USA). The measurements were performed from −40 to 130 °C at a constant frequency of 1 Hz, and a heating rate of 3 °C·min^−1^. Five replicates with dimensions of 35 mm in length, 12 mm in width, and 3 mm in thickness were tested for each type of composite. The storage moduli of the composites prepared under different modification conditions were analyzed.

The interfacial bonding between the wood fiber and the polymer matrix can be evaluated using the adhesion factor (*A*), which is determined from DMA data at 40 °C based on the study of Kubat et al. [30] by using Equation (2):

(2) $$A=\left(1/\left(1-V_{f}\right)\right)\left(\tan\delta_{c}/\tan\delta_{m}\right)-1$$

where c and m subscripts denote composite and matrix, respectively, and *V*_f_ is the fiber volume fraction. A low value of *A* is an indicator of good adhesion or high degree of interaction between the two phases and vice versa. 

#### 2.5.2. Scanning Electron Microscopy (SEM)

Morphologies of the wood fiber/HDPE composites were analyzed by a QUANTA 200 electron microscope (FEI Company, Eindhoven, The Netherlands) at the 300× magnification. To study the micro-morphology, the specimens were completely frozen in liquid nitrogen to impede the plastic deformation of the matrix and to obtain a well-defined fiber–matrix interface. A cross-section with a thickness lower than 3 mm was selected for scanning electron microscopy (SEM) investigations. The samples were coated with platinum prior to the observation to improve the surface conductivity, and observed at an acceleration voltage of 15 kV. The samples were mounted on the aluminum sample holder and placed in the specimen chamber in a vacuum condition of 0.06 mbar at room temperature.

## 3. Results and Discussion

### 3.1. Range Analysis

The range analysis method is based on calculating the range (*R* value) of each column in the orthogonal Table 2 through statistical methods. This method can be used to determine the major and minor orders between the processing parameters, in order to obtain the optimum combination of levels. For determining the optimum combination, we assumed that a greater range corresponds to a factor with a greater impact. When constructing the optimum combination of levels, the δ values determine the pros and cons of each factor. The calculation results are listed in Table 3. With respect to the impact strength of the prepared WPCs, the *R* value corresponding to the steam temperature, steam pressure, and processing time are 4.08, 1.42, and 0.18, respectively. Clearly, the *R* value corresponding to the steam temperature is the maximum, and it is the minimum for the processing time, and the results were consistent with those of the tensile and flexural strength of the prepared WPCs. Thus, the following major and minor relationships between the processing parameters and the mechanical properties of the prepared WPCs exit: the steam temperature (*A*) is predicted to have the strongest effect; the steam pressure (*B*) has the second-strongest effect; and the processing time (*C*) has the weakest effect on the mechanical properties.

**Figure 1:**
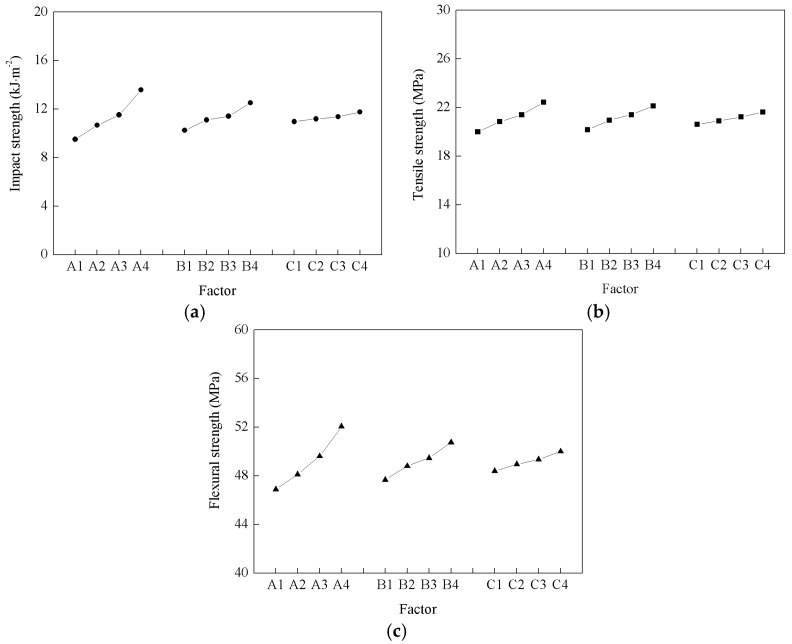
Range analysis of the effect of the process parameters on the impact, tensile, and flexural strengths of the wood fiber/high-density polyethylene (HDPE) composites. (**a**) Range analysis of wood plastic composite (WPC) impact; (**b**) Range analysis of WPC tensile; (**c**) Range analysis of WPC flexural.

The orthogonal design method strives to estimate the overall experimental results from a small number of experiments. Therefore, the optimum result may occur for a parameter combination not used in the experiments. In order to determine the best result that might exist, a level trend analysis was performed on the simulated results. The level trends obtained for the different factors are illustrated in Figure 1, which indicate that increasing the temperature, the steam pressure, and the processing time can all enhance the mechanical properties of the WPCs, with the modification temperature exhibiting the strongest effect, followed by the steam pressure and the processing time. According to the trends shown in Figure 1, the theoretical optimal combination is *A*4*B*4*C*4. Through comprehensively analyzing the relationship between the modification temperature, the steam pressure, and the processing treatment, the following optimal processing conditions were obtained for the high temperature and pressurized steam modification of wood fibers: a steam temperature of 170 °C, a steam pressure of 0.8 MPa, and a processing time of 20 min. 

### 3.2. Variance Analysis

The results of variance analysis are listed in Table 4. The analysis of the variance indicated that the order of the impact of the three factors is *A* > *B* > *C*, which is consistent with the results of the range analysis, objectively verifying the rationality of the variance analysis [31]. The maximum differences in S of the three factors were 0.03, 0.02, and 0.06, respectively, with errors of only 2.6%, 0.32%, and 0.16%, compared to the variance values. The small errors indicated that the level step selection and the experimental error of each factor did not have a large influence and that the range analysis and the variance analysis basically yielded consistent results. Therefore, the range analysis objectively verifies the rationality of the variance analysis.

### 3.3. Significance Analysis

In order to determine the impact of the different factors, a significance analysis was carried out [32]. According to mathematical statistics, the *p* value is considered as the significance probability. When *p* < 0.05, a factor is considered having a significant effect on the experimental results. The values listed in Table 5, Table 6 and Table 7 show that the significance probabilities of *A* and *B* are only slightly higher than 0.05, with *p*(*A*) < *p*(*B*). This indicates that factor *A* (the modification temperature) has the strongest effect on the mechanical properties of WPCs, followed by factor *B* (the steam pressure). The significance of factor *C* (the processing time) is not obvious. The results of the significance analysis are consistent with the results of the range and variance analyses, which indicate that the selected levels in the orthogonal design plan are reasonable and that the error is also reasonable at a certain level. 

The mechanical properties of the wood fiber/HDPE composites were enhanced due to the fact that through high temperature and pressurized steam treatment, the surface roughness of the wood fibers increased, the polarity on the fibers decreased, the in-depth diffusion and the mechanical interlock capability enhanced, and the interfacial compatibility between the wood fibers and the plastic matrix improved. The effect of the modification treatment increased with the increase of the modification temperature, pressure, and processing time. Compared to the WPCs prepared from untreated fibers, the WPCs prepared from fibers modified at 170 °C and 0.8 MPa for 20 min showed 20.17%, 18.5%, and 19.3% increases in tensile, bending, and impact strengths, respectively. In the high temperature and pressurized steam equipment, the operation for increasing temperature and pressure is simple and rapid, the energy cost is relatively low, and the enhancement in mechanical properties of WPCs is significantly high. Therefore, the modification condition combination can be considered as a better modification scheme with the advantages of low cost and high benefit.

### 3.4. Effect of the High Temperature and Pressurized Steam Treatment on the Hollocellulose Content of the Wood Fibers

**Figure 2:**
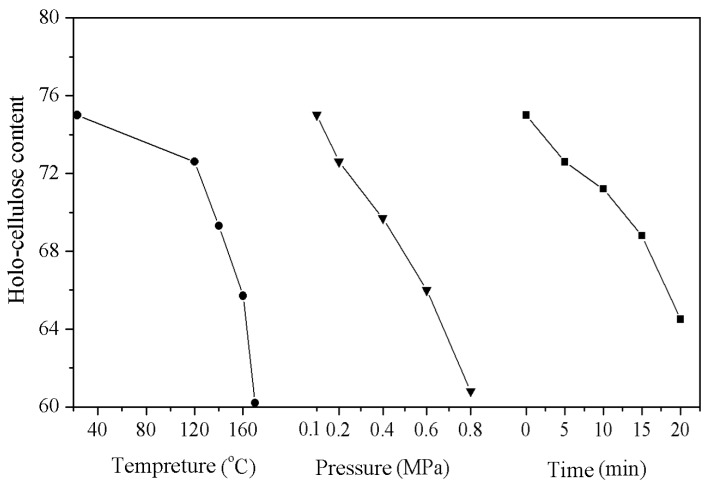
Effect of high temperature and pressurized steam on the hollocellulose content of wood fibers.

Figure 2 shows the hollocellulose content of wood fibers as a function of steam temperature, pressure, and retention time. The hollocellulose content of the wood fibers was found to decrease with increasing temperature and pressure because the high-temperature and high-pressure steam could remove the acetyl group of the semi-cellulose and induce the formation of acetic acid. Under the resulting acidic conditions, the glycoside bond breaks and a more thorough degradation occurs, whereas the lignin only experiences a slight softening rather than degradation due to its higher thermal stability. 

### 3.5. Effect of Steam Treatments on pH of Wood Fibers

**Figure 3:**
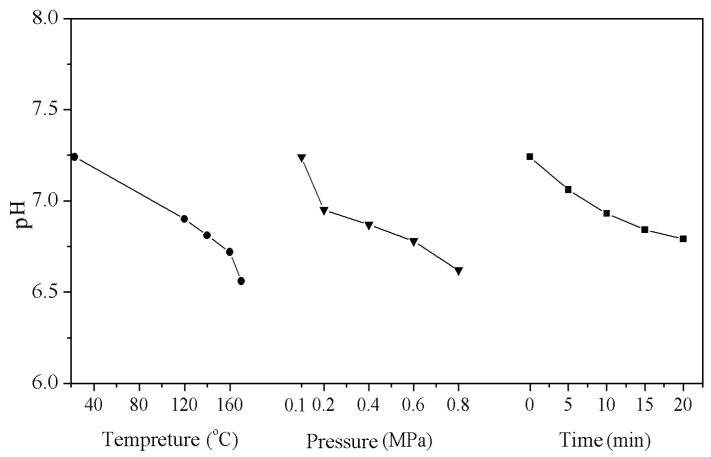
Effect of the high temperature and pressurized steam on the pH of the wood fibers.

The results of pH of wood fibers under various steam treatments are shown in Figure 3. The pH of untreated wood fibers (control sample) was 7.25. After high temperature and pressurized steam treatments, the pH of wood fibers decreased remarkably. The pH of the treated fibers under 160 °C for 15 min was reduced to 6.5 from 7.25 of the control sample. Moreover, the pH of the fibers decreased with increasing temperature, pressure, and retention time of the treatment. This result indicated that the acidic properties of wood fibers increased after steam treatment. It was also observed that the color of the modified fibers became more intense [33]. During the treatment, the semi-cellulose with its comparatively poor heat resistance partially degrades, losing its acetyl group, resulting in the formation of acetic acid. During the modification process, an increase in either temperature, pressure, or processing time leads to the generation of a higher amount of acidic substances and, thereby, a higher acidity. 

### 3.6. Fourier Transform Infrared Analysis

**Figure 4:**
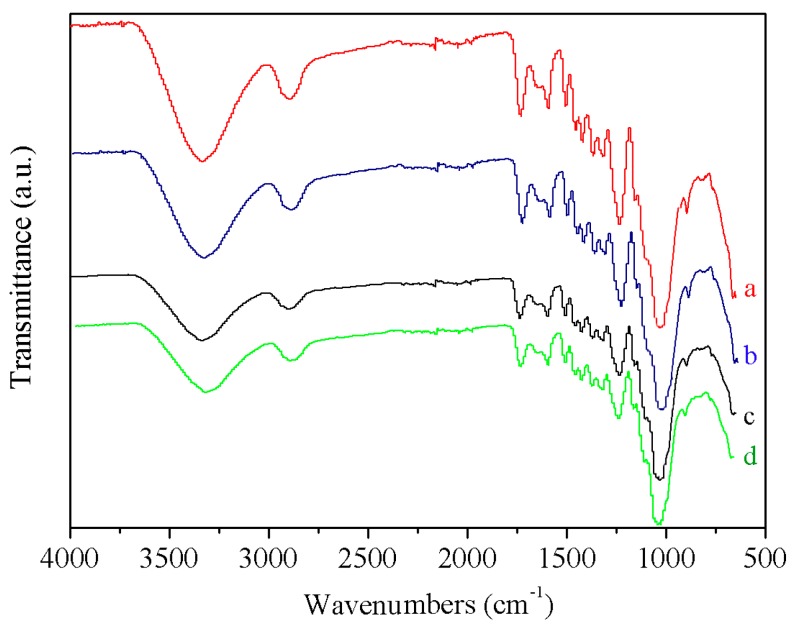
Comparison of the fourier transform infrared (FTIR) spectra obtained for untreated wood fibers and wood fibers modified through a high temperature and pressurized steam treatment under different conditions: a: untreated fibers; b: *T* = 120 °C, *p* = 0.2 MPa, *t* = 5 min; c: *T* = 160 °C, *p* = 0.6 MPa, *t* = 15 min; and d: *T* = 170 °C, *p* = 0.8 MPa, *t* = 5 min.

The surface infrared spectra for the untreated wood fibers and the wood fibers modified through high temperature and pressurized steam treatment are shown in Figure 4. Compared to the untreated wood fibers (Figure 4a), the intensities of the adsorption peaks centered at 3430 cm^−1^ (–OH) and 1738 cm^−1^ (C=O) [34] decreased for the treated wood fibers. The intensity of the hydroxyl group absorption peak showed obvious decrease. Owing to the exposure to the high-temperature and high-pressure steam, a “bridging” reaction might occur between pairs of free hydroxyl groups. This resulted in the formation of an ether bond and a reduced number of free hydroxyl groups. Moreover, the chemical structure of the semi-cellulose was subjected to severe degradation. During degradation, in the presence of a certain amount of adsorbed water, the number of carbonyl groups (C=O) decreases because the acetyl groups on the polysaccharide chain can easily hydrolyze to form acetic acid. When either the steam temperature, steam pressure, or the modification time was increased, the intensity of the C=O adsorption peak significantly declined. Furthermore, the intensities of the adsorption peaks at 1424 cm^−1^ (CH_2_ vibration) [35], 1375 cm^−1^ (CH bending vibration) [36], 1159 cm^−1^ (C–O–C stretching vibration), and 1055 cm^−1^ (C–O stretching vibration) [37] were all found to slightly decrease.

### 3.7. X-ray Diffraction Analysis

**Figure 5:**
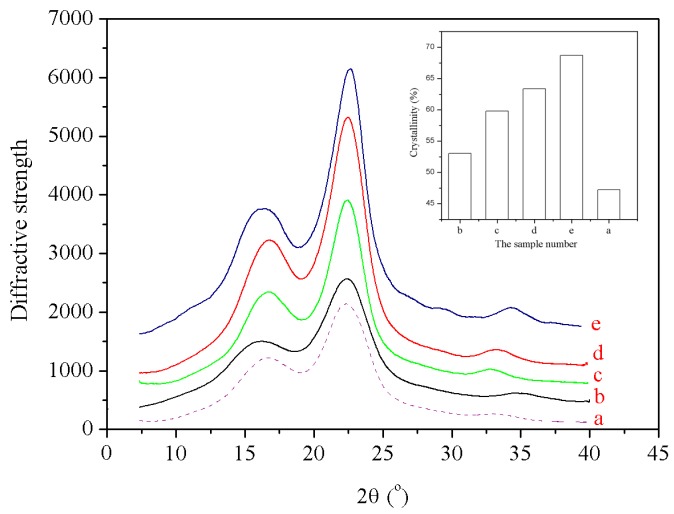
X-ray diffraction (XRD) patterns obtained for the untreated wood fibers and the wood fibers modified through a high temperature and pressurized steam treatment under different conditions: a: untreated fibers; b: *T* = 120 °C, *p* = 0.2 MPa, *t* = 5 min; c: *T* = 160 °C, *p* = 0.6 MPa, *t* = 5 min; d: *T* = 140 °C, *p* = 0.6 MPa, *t* = 15 min; and e: *T* = 170 °C, *p* = 0.8 MPa, *t* = 5 min.

The crystallinity of wood materials is one of the primary factors affecting their strength and mechanical properties. Figure 5 illustrates the X-ray diffraction (XRD) patterns obtained for the untreated and modified wood fibers [38]. Figure 5 and Table 8 show that the (002) diffraction peaks recorded for the untreated and the modified wood fibers are both centered at around 22° (ranging from 21.32° to 22.88°). This result indicates that the modification process resulted in a limited change of the crystalline regions of wood fibers, exhibiting an insignificant effect on the distance between the crystal layers. However, the modification process had a profound impact on the degree of crystallinity of the wood fibers, as shown in Table 8.

Figure 5 demonstrates that, compared to the untreated wood fibers, the crystallinity of the modified wood fibers gradually increased with the steam temperature, steam pressure, and the processing time. The untreated wood fibers had a crystallinity of 46.68%. The crystallinity of the modified wood fibers increased significantly when they were exposed to high temperature and pressurized steam. The crystallinity of the treated fibers under 170 °C and pressure of 0.8 MPa for 5 min was increased to 68.94% from 46.68% corresponding to the control sample, showing an increase in amplitude by 31.2%. During the steam treatment, the hydroxyl groups in the quasi-crystalline amorphous regions of the cellulose molecular chains can react with each other to form ether bonds, which induce the micro-fibrils to arrange in a more orderly fashion, move close to the crystallized regions and re-orientate. Therefore, high-temperature and pressurized steam modification can be used to increase the crystallinity of wood fibers, and the crystallinity increases with both the temperature and the pressure.

### 3.8. Thermal Analysis

**Figure 6:**
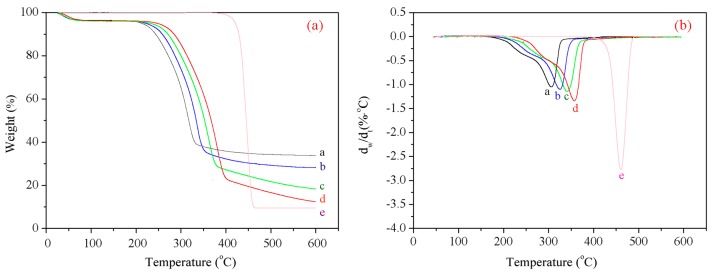
(**a**) TG and (**b**) DTG curves obtained for the unmodified and modified wood fibers; a: untreated fibers; b: *T* = 120 °C, *p* = 0.2 MPa, *t* = 5 min; c: *T* = 170 °C, *p* = 0.8 MPa, *t* = 5 min; d: *T* = 170 °C, *p* = 0.8 MPa, *t* = 15 min; and e: HDPE.

Figure 6a,b show the themogravimetric (TG) and derivative thermal gravimetric (DTG) curves for the untreated and treated wood fibers, respectively. Figure 6 and Table 9 show that when the temperature increases from room temperature to 600 °C, the pyrolysis of the wood fibers undergoes three stages, with the second stage (210–400 °C) being the main pyrolysis stage. 

Compared to the untreated fibers, the wood fibers exposed to the high temperature and pressurized steam showed a higher pyrolysis peak temperature, i.e., a better heat stability. When the reaction temperature was 200 °C or higher, the required pyrolysis temperature and the weight loss rate both increased with the temperature, pressure, and/or the processing time. When subjected to high temperature and pressurized steam with high moisture content, parts of the structure of the hemicellulose reorganized, which resulted in a slight increase in lignin content. Notably, the methoxy group, one of the characteristic functional groups of lignin, is relatively stable; therefore, the increased lignin content enhances the thermal stability of the wood fibers [39]. Furthermore, the hydroxyl groups in the cellulose can get oxidized into relatively stable aldehyde, carbonyl, and carboxyl groups to form oxidized cellulose. Therefore, a higher steam temperature and a greater pressure resulted in a better heat stability.

### 3.9. Dynamic Mechanical Analysis

**Figure 7:**
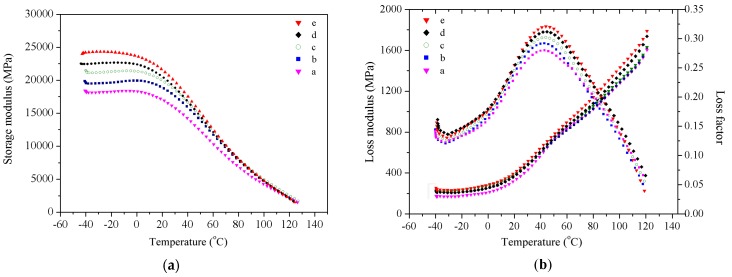
Variation of (**a**) the storage modulus and (**b**) the loss modulus and the loss factor with the steam temperature: a: untreated fibers; b: *T* = 120 °C, *p* = 0.2 MPa, *t* = 5 min; c: *T* = 160 °C, *p* = 0.6 MPa, *t* = 5 min; d: *T* = 160 °C, *p* = 0.6 MPa, *t* = 15 min; and e: *T* = 170 °C, *p* = 0.8 MPa, *t* = 5 min.

The storage moduli of wood fiber/HDPE composites at different steam treatment conditions are shown in Figure 7a. The storage modulus of the WPCs increased with the steam temperature, steam pressure, and processing time. Compared to the WPCs prepared from untreated wood fibers, the storage moduli of the WPCs prepared from modified fibers showed a higher increase in amplitude. When the fibers were modified through exposure to steam at a temperature of 170 °C and a pressure of 0.8 MPa for 20 min, the resulting WPCs showed the highest storage moduli.

Figure 7b reveals the variation of the loss modulus and the loss factor of the prepared WPCs as a function of temperature. The peaks in these two curves have been used to identify the glass-transition temperature (*T*_g_), and use the temperature corresponding to the maximum loss modulus as *T*_g_, which corresponds to the temperature at which the polymer chain begins to move or freeze [40]. In this experiment, the increase of *T*_g_ indicates that the length of the HDPE resin molecular chains is limited and that the high temperature and pressurized steam modification can improve the compatibility between the wood fibers and the HDPE resin. Figure 7b demonstrates that the loss modulus curves obtained for the WPCs prepared from modified fibers are all single-peak curves, indicating that the modification leads to a good compatibility between the wood fibers and the HDPE. When the processing temperature was 170 °C, the steam pressure was 0.8 MPa, and the modification time was 5 min, the loss modulus of the WPCs prepared from the modified fibers acquired the maximum value.

As mentioned above, the interfacial bonding between the wood fibers and the polymer matrix can also be evaluated using the adhesion factor (*A*). Strong interactions between the fibers and polymer matrix at the interface tend to reduce the macromolecular mobility in the vicinity of the filler surface compared to that in the bulk matrix [41,42]. The *A* values for the WPCs at 40 °C are calculated by using Equation (2). The WPCs prepared from unmodified fibers exhibited the highest *A* values of 0.757 (the weakest interfacial interaction); however, the WPCs prepared from fibers modified by high temperature and pressurized steam treatment (170 °C, 0.8 MPa, 20 min) exhibited the strongest interactions (lowest *A* value: 0.486). This result demonstrated that high temperature and pressurized steam strongly affected the interfacial bonding between the wood fibers and the polymer matrix, and also the properties of the prepared composites.

### 3.10. Analysis of the Micro-Morphology 

**Figure 8:**
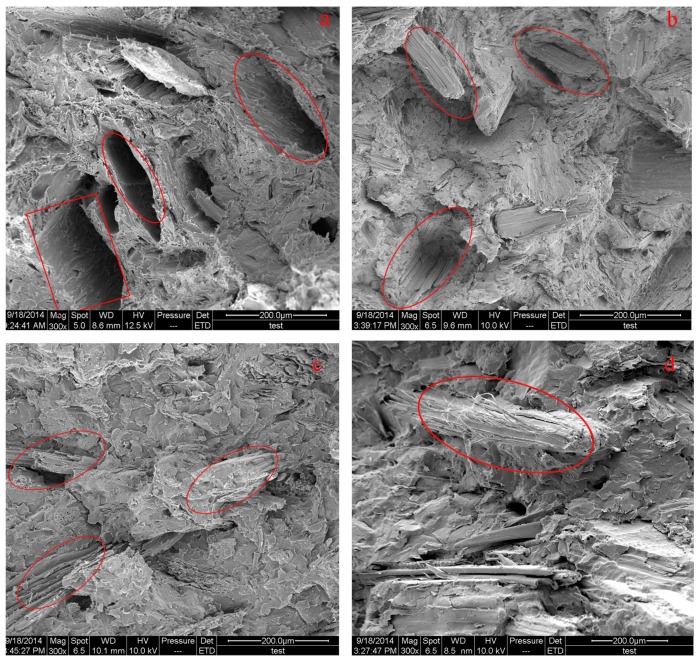
Scanning electron microscopy (SEM) micrographs of WPC prepared from untreated wood fibers and fibers modified through high-temperature high-pressure steam under different conditions: (**a**) untreated fibers; (**b**) *T* = 120 °C, *p* = 0.2 MPa, *t* = 5 min; (**c**) *T* = 170 °C, *p* = 0.8 MPa, *t* = 5 min; and (**d**) *T* = 170 °C, *p* = 0.8 MPa, *t* = 15 min.

Figure 8 shows SEM micrographs of wood fiber/HDPE composites prepared from the wood fibers modified by exposure to high temperature and pressurized steam. Figure 8a shows that the fractured surface of the WPCs prepared from untreated fibers is not smooth. The pull-out of fibers occurred more frequently, which resulted in a large number of voids on the fractured surface. The interfaces between the plastic matrix and the wood fibers are clear, with obvious signs of agglomeration. When the fibers were modified through exposure to steam at a temperature of 120 °C and a pressure of 0.2 MPa for 5 min, the polarity of the fibers decreased, which contributed to the improved interfacial bonding between wood fibers and plastic matrix. Although the pull-out was not completely suppressed and voids still existed on the fractured surface, the interfacial bonding was better compared to the composites prepared from untreated fibers, as illustrated in Figure 8b. Clearly, the interfacial bonding between the steam-modified wood fibers and the plastic matrix significantly improved with the increase of steam temperature and pressure. Therefore, the pull-out phenomenon was hardly observed on the fractured surface; however, the pulling fracture phenomena occurred instead. The pulling fracture phenomena led to an increased number of holes, smaller holes, and a rougher surface. However, when the fibers were modified through exposure to steam at a temperature of 120 °C and a pressure of 0.8 MPa for 5 min, fibrils were found on the surface of the modified wood fibers. The interface between the wood fibers and the resin matrix was relatively vague; the remaining empty slots were smaller and more irregular after the fibers were pulled out. These results indicated that the prepared WPCs showed better mechanical properties, as revealed in Figure 8d.

## 4. Conclusions

Adopting the orthogonal design method, the mechanism of high temperature and pressurized steam modification of wood fibers and the effect of the modification process on the performance of composite materials fabricated from the modified fibers were investigated in this study. The results can be summarized as follows:

It proved feasible to apply the orthogonal design method to the preparation of WPCs from fibers modified by high temperature and pressurized steam. The orthogonal design method could be effectively used to optimize the processing parameters. The results of the variance, range, and significance analysis were consistent with the SEM and DMA results. The steam temperature was found to have the greatest effect on the mechanical properties of WPCs, followed by the steam pressure. A temperature of 170 °C, a vapor pressure of 0.8 MPa, and processing time of 20 min were determined as the optimum parameters. Compared to the WPCs prepared from untreated fibers, the tensile, bending, and impact strengths of the WPCs prepared from modified fibers increased by up to 20.17%, 18.5%, and 19.3%, respectively.

When exposed to a high temperature and pressurized steam treatment, the number of hydroxyl groups and the surface polarity of the wood fibers decreased; however, the crystallinity of wood fibers increased. When the processing temperature, pressure, and time were 170 °C, 0.8 MPa, and 20 min, respectively, the crystallinity of the cellulose increased to 62.95%, with a 31.2% increase in magnitude compared to the untreated wood fibers. With increasing steam temperature, pressure, and processing time, the thermal stability, storage moduli of the prepared WPCs, and the mechanical properties of the wood fiber significantly improved; however, the hollocellulose content and the pH of wood fibers gradually decreased.

## Figures and Tables

**Table 1:** Factors and levels of the orthogonal array.

Number	Factors	Levels			
		1	2	3	4
1	Temperature (°C)	120	140	160	170
2	Steam pressure (MPa)	0.2	0.4	0.6	0.8
3	Time (min)	5	10	15	20

**Table 2:** Orthogonal experimental design for the L16 (45) orthogonal array.

Piece Number	Temperature (°C)	Steam Pressure (MPa)	Time (min)	Test Battery
	*A*	*B*	*C*	
1	120	0.2	5	*A*1*B*1*C*1
2	120	0.4	10	*A*1*B*2*C*2
3	120	0.6	15	*A*1*B*3*C*3
4	120	0.8	20	*A*1*B*4*C*4
5	140	0.2	10	*A*2*B*1*C*2
6	140	0.4	5	*A*2*B*2*C*1
7	140	0.6	20	*A*2*B*3*C*4
8	140	0.8	15	*A*2*B*4*C*3
9	160	0.2	15	*A*3*B*1*C*3
10	160	0.4	20	*A*3*B*2*C*4
11	160	0.6	5	*A*3*B*3*C*1
12	160	0.8	10	*A*3*B*4*C*2
13	170	0.2	20	*A*4*B*1*C*4
14	170	0.4	15	*A*4*B*2*C*3
15	170	0.6	10	*A*4*B*3*C*2
16	170	0.8	5	*A*4*B*4*C*1

**Table 3:** Results of the range analysis of the effect of the processing parameters on the mechanical properties of the prepared wood plastic composites (WPCs).

	Impact Strength (kJ·m^−2^)			Tensile (MPa)			Flexural (MPa)		
	Temperature	Pressure	Time	Temperature	Pressure	Time	Temperature	Pressure	Time
δ*_j_*_1_	38.04	41.02	43.87	80.04	80.70	83.64	187.59	190.71	193.58
δ*_j_*_2_	42.68	44.41	44.77	83.36	83.80	83.63	192.49	195.23	195.81
δ*_j_*_3_	46.10	45.65	45.49	85.59	85.65	84.93	198.47	197.87	197.39
δ*_j_*_4_	54.35	50.09	47.04	89.72	88.56	86.51	208.28	203.02	200.05
$\bar{\delta_{j1}}$	9.51	10.26	10.97	20.01	20.18	20.91	46.90	47.68	48.40
$\bar{\delta_{j2}}$	10.67	11.10	11.19	20.84	20.95	20.91	48.12	48.81	48.95
$\bar{\delta_{j3}}$	11.53	11.41	11.37	21.40	21.41	21.23	49.62	49.47	49.35
$\bar{\delta_{j4}}$	13.59	12.52	11.76	22.43	22.14	21.63	52.07	50.76	50.01
*R*	4.08	1.42	0.18	2.42	1.19	0.33	5.17	1.95	0.40
Flexural	*A* > *B* > *C*			*A* > *B* > *C*			*A* > *B* > *C*		

**Table 4:** Variance analysis of the impact, tensile, and flexural strengths of the wood fiber/HDPE composites.

	Impact Strength (kJ·m^−2^)			Tensile Strength (MPa)			Flexural Strength (MPa)		
	*A* × 10^3^	*B* × 10^3^	*C* × 10^3^	*A* × 10^3^	*B* × 10^3^	*C* × 10^3^	*A* × 10^3^	*B* × 10^3^	*C* × 10^3^
$\delta_{j1}^{2}$	1.45	1.68	1.92	6.41	6.51	7	35.19	36.37	37.47
$\delta_{j2}^{2}$	1.82	1.97	2.01	6.95	7.02	6.99	37.05	38.11	38.34
$\delta_{j3}^{2}$	2.23	2.08	2.07	7.33	7.34	7.21	39.39	39.15	38.92
$\delta_{j4}^{2}$	2.95	2.51	2.21	8.05	7.84	7.48	43.38	41.22	40.02
*P*	8.35	8.25	8.21	28.73	28.71	28.69	155.01	154.85	154.79
*Q*	2.09	2.06	2.05	7.18	7.18	7.17	38.75	38.71	38.69
*S*	1.17	1.15	1.14	6.27	6.26	6.25	37.84	37.8	37.78
Impact	*A* > *B* > *C*			*A* > *B* > *C*			*A* > *B* > *C*		

$P=\delta_{j1}^{2}+\delta_{j2}^{2}+\delta_{j3}^{2}+\delta_{j4}^{2}$; *Q* = $\frac{P}{4}$; $S=Q-\frac{{\left(\sum_{i=1}^{16}\delta_{i}\right)}^{2}}{16}$; δ*_i_* is the experimental indicator of each simulation (*i* = 1, …, 16).

**Table 5:** Significance analysis for the factors affecting the impact strength.

Factor	Degree of Freedom	Sum of Deviation Square	Mean Square Error	*F*	*p*	Significance
*A*	3	35.53	11.84	59.82	0.000073	***
*B*	3	10.54	3.51	17.75	0.00218	**
*C*	3	1.35	0.45	2.27	0.18	
Error	6	0.0003	0.00110	–	–	
Model	15	0.0170	0.00005	–	–	

“–” means not significant, *p* > 0.05; “**”means very significant, 0.001 < *p* < 0.01; “***” means highly significant, *p* < 0.001.

**Table 6:** Significance analysis for the factors affecting the tensile strength.

Factor	Degree of Freedom	Sum of Deviation Square	Mean Square Error	*F*	*p*	Significance
*A*	3	12.38	4.13	13.83	0.004194	**
*B*	3	8.15	2.72	9.11	0.01186	*
*C*	3	1.40	0.47	1.56	0.01186	*
Error	6	0.0003	0.00110	–	–	
Model	15	0.0170	0.00005	–	–	

“–” means not significant, *p* > 0.05; “*”means significant, 0.01 < *p* < 0.05; “**”means very significant, 0.001 < *p* < 0.01.

**Table 7:** Significance analysis for the factors affecting the flexural strength.

Factor	Degree of Freedom	Sum of Deviation Square	Mean Square Error	*F*	*p*	Significance
*A*	3	59.49	19.83	57.26	0.000083	***
*B*	3	19.84	6.61	19.10	0.0018	**
*C*	3	5.56	1.85	5.53	0.047	*
Error	6	0.0003	0.00110	–	–	
Model	15	0.0170	0.00005	–	–	

“–” means not significant, *p* > 0.05; “*”means significant, 0.01 < *p* < 0.05; “**”means very significant, 0.001 < *p* < 0.01; “***” means highly significant, *p* < 0.001.

**Table 8:** Comparison of the crystal properties and degrees of crystallinity of the unmodified and modified wood fibers.

Category	Piece Number	002 Crystal Plane Angle	Crystallinity
High temperature and pressurized steam treatment	b	22.62°	54.02%
	c	22.82°	58.80%
	d	22.79°	52.74%
	e	21.67°	68.94%
Control sample	a	22.88°	46.68%

**Table 9:** Comparison of the pyrolysis characteristics of the unmodified and modified wood fibers.

Material Code	TGA Analysis					
	First Stage		Second Stage		Third Stage	
	Temperature Range	Weight Loss Ratio	Temperature Range	Weight Loss Ratio	Temperature Range	Weight Loss Ratio
a	30%–205%	4.4%	205%–315%	56%	315%–600%	9.3%
b	30%–220%	4.7%	220%–340%	59%	340%–600%	6.6%
c	30%–230%	4.6%	230%–350%	64%	350%–600%	8.5%
d	30%–250%	4.6%	250%–370%	66%	370%–600%	7.3%
e	0%	0%	0%	0%	375%–475%	91%
